# Protein Profiling of WERI-RB1 and Etoposide-Resistant WERI-ETOR Reveals New Insights into Topoisomerase Inhibitor Resistance in Retinoblastoma

**DOI:** 10.3390/ijms23074058

**Published:** 2022-04-06

**Authors:** Vinodh Kakkassery, Timo Gemoll, Miriam M. Kraemer, Thorben Sauer, Aysegül Tura, Mahdy Ranjbar, Salvatore Grisanti, Stephanie C. Joachim, Stefan Mergler, Jacqueline Reinhard

**Affiliations:** 1 Department of Ophthalmology, University of Luebeck, Ratzeburger Allee 160, 23538 Luebeck, Germany; ayseguel.tura@uksh.de (A.T.); mahdy.ranjbar@uksh.de (M.R.); salvatore.grisanti@uksh.de (S.G.); 2 Section for Translational Surgical Oncology and Biobanking, Department of Surgery, University of Luebeck and University Hospital Clinic Schleswig-Holstein, Ratzeburger Allee 160, 23538 Luebeck, Germany; timo.gemoll@uni-luebeck.de (T.G.); thorben.sauer@student.uni-luebeck.de (T.S.); 3 Department of Cell Morphology and Molecular Neurobiology, Faculty of Biology and Biotechnology, Ruhr-University Bochum, Universitaetsstraße 150, 44780 Bochum, Germany; m.kraemer@mmk2.de; 4 Experimental Eye Research Institute, University Eye Hospital, Ruhr-University Bochum, In der Schornau 23-25, 44892 Bochum, Germany; stephanie.joachim@rub.de; 5 Department of Ophthalmology, Charité-Universitaetsmedizin Berlin, Corporate Member of Freie Universitaet Berlin, Humboldt-Universitaet zu Berlin, Augustenberger Platz 1, 13353 Berlin, Germany; stefan.mergler@charite.de

**Keywords:** chemotherapy resistance, mass spectrometry, retinoblastoma, WERI-ETOR, WERI-RB1

## Abstract

Chemotherapy resistance is one of the reasons for eye loss in patients with retinoblastoma (RB). RB chemotherapy resistance has been studied in different cell culture models, such as WERI-RB1. In addition, chemotherapy-resistant RB subclones, such as the etoposide-resistant WERI-ETOR cell line have been established to improve the understanding of chemotherapy resistance in RB. The objective of this study was to characterize cell line models of an etoposide-sensitive WERI-RB1 and its etoposide-resistant subclone, WERI-ETOR, by proteomic analysis. Subsequently, quantitative proteomics data served for correlation analysis with known drug perturbation profiles. Methodically, WERI-RB1 and WERI-ETOR were cultured, and prepared for quantitative mass spectrometry (MS). This was carried out in a data-independent acquisition (DIA) mode. The raw SWATH (sequential window acquisition of all theoretical mass spectra) files were processed using neural networks in a library-free mode along with machine-learning algorithms. Pathway-enrichment analysis was performed using the REACTOME-pathway resource, and correlated to the molecular signature database (MSigDB) hallmark gene set collections for functional annotation. Furthermore, a drug-connectivity analysis using the L1000 database was carried out to associate the mechanism of action (MOA) for different anticancer reagents to WERI-RB1/WERI-ETOR signatures. A total of 4756 proteins were identified across all samples, showing a distinct clustering between the groups. Of these proteins, 64 were significantly altered (q < 0.05 & log2FC |>2|, 22 higher in WERI-ETOR). Pathway analysis revealed the “retinoid metabolism and transport” pathway as an enriched metabolic pathway in WERI-ETOR cells, while the “sphingolipid de novo biosynthesis” pathway was identified in the WERI-RB1 cell line. In addition, this study revealed similar protein signatures of topoisomerase inhibitors in WERI-ETOR cells as well as ATPase inhibitors, acetylcholine receptor antagonists, and vascular endothelial growth factor receptor (VEGFR) inhibitors in the WERI-RB1 cell line. In this study, WERI-RB1 and WERI-ETOR were analyzed as a cell line model for chemotherapy resistance in RB using data-independent MS. Analysis of the global proteome identified activation of “sphingolipid de novo biosynthesis” in WERI-RB1, and revealed future potential treatment options for etoposide resistance in RB.

## 1. Introduction

To date, retinoblastoma (RB) has an incidence of 1/15,000 to 1/20,000 live births, and around 9000 new cases are diagnosed worldwide every year [1,2,3,4]. This makes it the most common malignant pediatric ocular tumor globally. Due to an improvement in therapeutic strategies in recent years, the survival rate in western countries is almost 99%, while death rates in Africa and Asia are still high [5,6,7]. Unfortunately, some affected eyes still need to be removed due to chemotherapy failure, which is also due to chemotherapy resistance [8].

Chemotherapy-resistance mechanisms in RB are unclear so far. To further investigate the mechanisms in RB, the WERI-RB1 cell line (Leibniz Institute-German Collection of Microorganisms and Cell Cultures, DSMZ No. ACC90) has been established by spontaneous outgrowth of an enucleated RB eye, as published by McFall et al. [9,10]. Among others, Busch et al. characterized the growth behavior of the WERI-RB1 cell line under cell-culture conditions [11]. To identify the mechanisms of chemotherapy resistance, the etoposide-resistant subclone WERI-ETOR was established by harvesting surviving cells after incubation with increasing etoposide doses from WERI-RB1, as described by Stephan et al. [12]. Previous studies also examined the parental WERI-RB1 and the etoposide-resistant subclone WERI-ETOR on the molecular level [13,14,15,16,17,18]. Furthermore, Busch et al. demonstrated a higher proliferation rate in WERI-ETOR as well as increased tumor formation and tumor size in an in vivo chick chorioallantoic-membrane assay [17]. Mergler et al. and Oronowicz et al. showed alterations in thermosensitive transient receptor potential channels in WERI-ETOR cells [13,16]. A publication by Kakkassery et al. proved an upregulation of sphingosine-1-phosphate in the WERI-ETOR cell line as a potential resistance mechanism in RB [14]. Recently, Reinhard et al. reported on changes of the extracellular matrix (ECM) in WERI-ETOR compared to WERI-RB1 cells [15].

Interestingly, the nature of the global proteome in RB remains unclear. While genotype–phenotype relationships are complex, proteins are generally known to be the effectors. However, molecular signatures associated with a phenotype of interest could also occur on a different level, e.g., as expression signatures and methylation signatures [19,20,21,22]. As such, proteins are suited to determine the signature of a physiological phenotype as well as the point of intervention for drug and health treatments. Hereby, LC-ESI-MS/MS in a data-independent (DIA/SWATH) mode plays a decisive role. DIA/SWATH uses variable mass windows to enable the complete measurement of all detectable proteins in a sample. This allows the identification and quantification of thousands of proteins in just one measurement. Compared to alternative relative quantification techniques, such as iTRAQ, label-free SWATH offers higher sensitivity, reliability, and robustness. Furthermore, sample preparation for labeled proteomics is more complex as it requires, e.g., the covalent binding of isobaric labels to the peptide analytes [23]. Therefore, the specific aim of this study was to characterize the differences between the cell lines WERI-RB1 and WERI-ETOR on the proteomic level. The data for this investigation was collected by quantitative mass spectrometry (MS) in DIA mode in combination with machine-learning algorithms for mass spectrometric and statistical evaluation. The generated quantitative proteome data was used to gain further insight into the functional annotation, including intracellular-signaling pathways that differ between WERI-RB1 and WERI-ETOR. Furthermore, a drug-connectivity analysis using the L1000 database was used to correlate the mechanism of action (MOA) for different anticancer reagents to WERI-RB1/WERI-ETOR signatures.

## 2. Results

### 2.1. Protein-Expression Profiles of WERI-RB1 and WERI-ETOR Cell Lines

We performed quantitative MS using a DIA mode to detect proteins differentially expressed in WERI-RB1 and WERI-ETOR cell lines. Using a neuronal network-based identification workflow (DIA-NN, [24]), 4756 protein groups were identified. All entries with more than 70% missing values (MV) were removed, resulting in 2.2% MVs. The remaining MVs were imputed via replacement by random draws from a normal distribution. A table containing all protein quantification values is included in Supplemental Table S1. MS data have been deposited to the ProteomeXchange Consortium via the PRIDE partner repository with the dataset identifier PXD030924.

**Figure 1:**
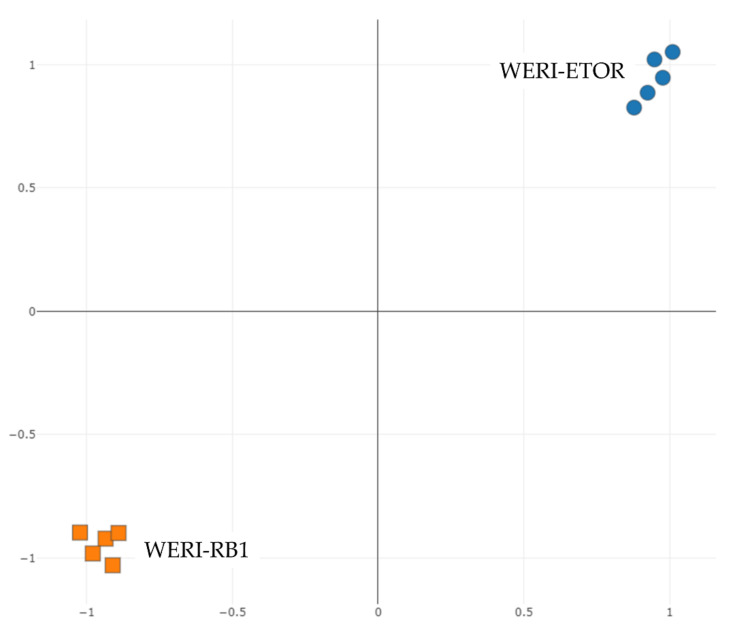
Unsupervised tSNE plot of WERI-RB1 (orange) and WERI-ETOR (blue) cell-line samples. The plot visualizes the close relationship with cell lines and the distinct clustering between sample groups (n = 5/group).

Phenotypic differences between both cell cultures were compared in a t-distributed stochastic neighbor embedding (tSNE) applying protein-expression data. A tSNE plot showed a clear differentiation of the analyzed groups (Figure 1).

**Figure 2:**
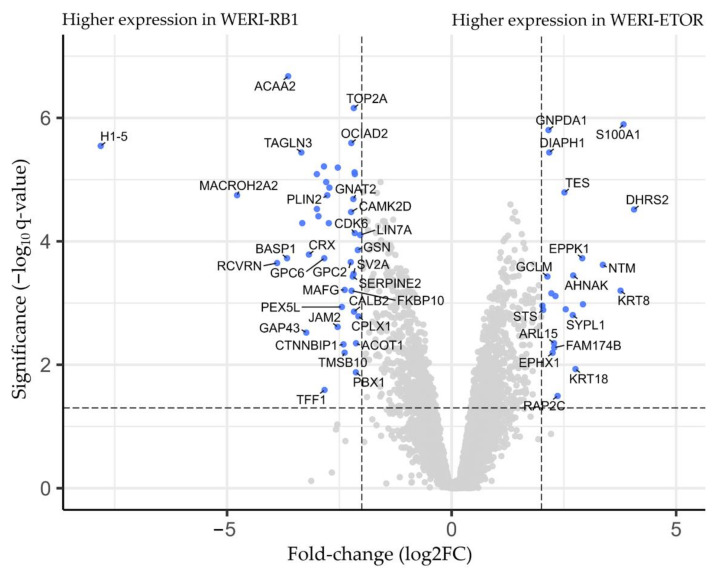
Volcano plot visualizing fold-change (*x*-axis) and statistical significance (*y*-axis). Proteins with a higher concentration in the WERI-ETOR cell line are presented on the right side. Proteins with a lower concentration in the WERI-ETOR cell line are displayed on the left side. All blue-marked proteins demonstrated a logarithmic fold-change (log2FC) of |>2| and a q-value < 0.05.

A total of 64 differentially expressed proteins (q < 0.05 & log2FC |>2|) were found between the two groups. Of these proteins, 22 (34%) showed a higher concentration, while 42 (66%) had a lower concentration in the WERI-ETOR cell line (Figure 2). The protein with the highest differential expression in WERI-ETOR was dehydrogenase/reductase 2 (DHRS2; q-value: 0.0000305 and log2FC: 4.06), whereas H1.5 linker histone, cluster member (H1–5) showed the highest differential expression in WERI-RB1 (q-value: 0.00000285 and log2FC: −7.81). A list of all differentially expressed proteins, including their corresponding log2FC and q values, is shown in Supplemental Table S2.

**Figure 3:**
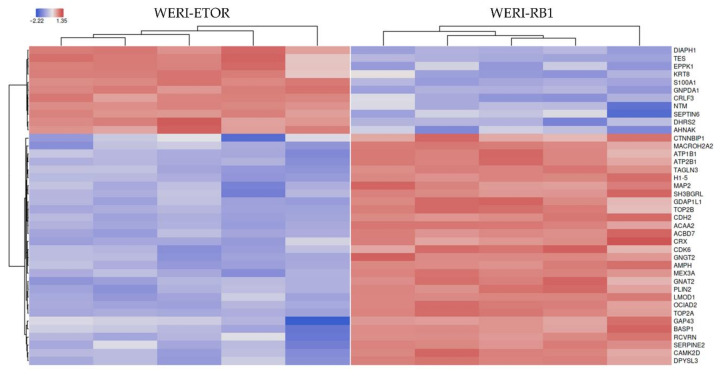
Heatmap of the top 40 selected features according to a cumulative ranking by the applied algorithms (LASSO, elastic nets, random forest, extreme gradient boosting). Red colors show high protein expression, and blue colors show low protein expression.

For validation of differential-expressed proteins, a feature importance calculation based on machine-learning algorithms (LASSO, elastic nets, random forest, extreme gradient boosting) selected the best possible proteins (n = 40) to predict group association (Table 1). What stands out in the table is that all top important features also showed statistical significance. Additionally, the most important proteins are displayed using a heatmap clustering (Figure 3).

### 2.2. Enrichment-Pathway Analysis of WERI-RB1 and WERI-ETOR Protein Signatures

**Figure 4:**
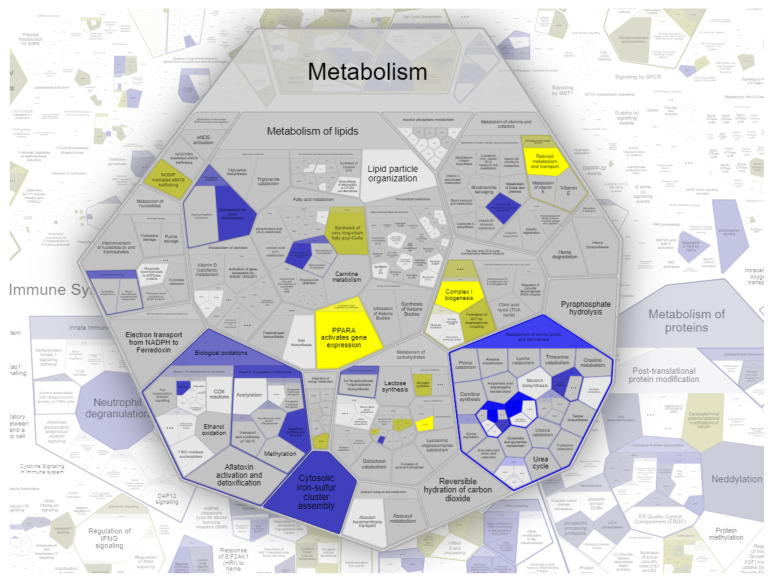
Voronoi diagram representation of the pathway “metabolism” by REACTOME analysis using all expressed protein elements of the WERI-RB1 and WERI-ETOR cell lines. Yellow colors show pathway activation in WERI-RB1 cells; blue colors show pathway activation in WERI-ETOR cells.

For a deeper insight into the different proteomes of the WERI-RB1 and WERI-ETOR cell lines, all proteins were uploaded to the REACTOME database. The REACTOME-pathway-enrichment algorithm was able to match 3934 out of 4756 (83%) proteins, which were associated with 2026 available pathways (submission date: May 2021). Among others, one of the most enriched metabolic pathways for the WERI-ETOR was “retinoid metabolism and transport” (*p* = 0.0029), while “sphingolipid de novo biosynthesis” (*p* = 0.033) was activated in WERI-RB1. Eleven identified proteins were represented in the network of “retinoid metabolism and transport”: retinol dehydrogenase 11 (RDH11), agrin (AGRN), N(G), N(G)-dimethylarginine dimethylaminohydrolase 1 (DDAH1), apolipoprotein E (APOE), syndecan-1 (SDC1), syndecan-2 (SDC2), glypican-2 (GPC2), apolipoprotein C-III (APOC3), apolipoprotein A-I (APOA1), syndecan-4 (SDC4), and glypican-6 (GPC6). The REACTOME network “sphingolipid de novo biosynthesis” contained eight identified proteins, namely aldehyde dehydrogenase family 3 member A2 (ALDH3A2), vesicle-associated membrane protein-associated protein A (VAPA), oxysterol-binding protein 1 (OSBP), serine palmitoyltransferase 1 (SPTLC1), sphingosine-1-phosphate lyase 1 (SGPL1), vesicle-associated membrane protein-associated protein B/C (VAPB), ceramide-transfer protein (CERT1), and 3-ketodihydrosphingosine reductase (KDSR). A Voronoi diagram representation of the pathway “metabolism” is shown in Figure 4.

### 2.3. Drug-Enrichment Analysis of WERI-RB1 and WERI-ETOR Protein Signatures

**Figure 5:**
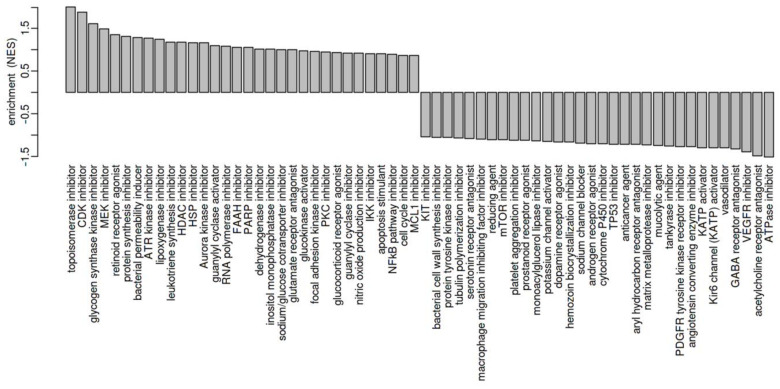
Visualization of the mechanism of action (MOA) across enriched drug profiles using the L1000 database. On the vertical axis, the GSEA normalized enrichment score of the MOA class is plotted.

To investigate if a certain drug-activity or drug-sensitivity signature matches our protein-expression data, a drug-connectivity analysis using the L1000 database was carried out. The most striking finding of these data was that the correlation between the etoposide-resistant cell line WERI-ETOR and the parental cell line WERI-RB1 revealed a very similar MOA for topoisomerase inhibitors, e.g., etoposide for the resistant cell line (Figure 5). These data demonstrate that topoisomerase inhibitors (most left) induce specific molecular changes, which are already active in WERI-ETOR cells. In turn, topoisomerase inhibitors lose their drug efficacy in these cell lines. ATPase inhibitors, acetylcholine receptor antagonists, and VEGFR inhibitors (most right) display the opposite MOA compared to WERI-ETOR cells and should thus overcome the developed therapy resistance.

## 3. Discussion

Drug resistance in RB is a clinical problem that can lead to the enucleation of a child’s eye to prevent distant metastasis. The present study was designed to characterize the RB cell line WERI-RB1 and its etoposide-resistant subclone WERI-ETOR at the global proteome level. The comparison of both cell lines identified 64 differentially expressed proteins (log2FC of |>2| and a q-value < 0.05; 22 higher and 42 lower expressed in WERI-ETOR). Pathway analysis by REACTOME algorithms revealed the most enriched metabolic pathway “retinoid metabolism and transport” for WERI-ETOR, while “sphingolipid de novo biosynthesis” was activated in WERI-RB1. These results support previous findings that linked RB samples with healthy biospecimens: Danda et al. identified a total of 3587 proteins using isobaric tags for relative and absolute quantitation (iTRAQ)-based quantitative mass spectrometric analysis and found 899 differentially expressed proteins between RB and healthy human retina samples [25]. Using the ingenuity pathway knowledge database, the top identified molecular and cellular processes were lipid metabolism, molecular transportation, small molecule biochemistry, nucleic acid metabolism as well as DNA replication, recombination, and repair [25]. Cheng et al., on the other hand, analyzed aqueous humor samples from RB patients and compared them with samples from patients without intraocular cancer using the comparative proteomic technique of iTRAQ coupled with offline two-dimensional liquid chromatography-tandem mass spectrometry. A total of 83 proteins were identified to be differentially expressed of which 44 were upregulated and 39 downregulated in the RB group [26]. Assisted by the DAVID bioinformatics resource, protein expression in RB has been implicated in endopeptidase inhibitory activity, peptidase inhibitor, enzyme inhibitor, serine-type endopeptidase inhibitor, structural molecule, lipid binding, and carbohydrate-binding activities [26]. Furthermore, in a different setting, Danda and colleagues performed proteomic analysis on human RB and healthy retina samples. A total of 3122 proteins were detected in this study, with 282 upregulated and 381 downregulated proteins in the RB sample [27]. Using the DAVID bioinformatics resource, the analysis revealed that most of the regulated proteins were primarily involved in glycoprotein, amyloid acute-inflammatory, and defensive responses. Furthermore, Naru et al. analyzed a human papilloma virus-positive RB sample, a human papilloma virus-negative RB sample, and healthy human retina tissue [28]. Collectively, 2828 proteins were identified in this study. While 78 proteins were differentially expressed between the human papilloma virus-positive RB sample and the human healthy control retina sample, 88 proteins showed a differential expression between the human papilloma virus-negative RB sample and the human healthy control retina tissue. Bioinformatic analysis performed with the PANTHER classification system showed that most of these proteins were involved in catalytic activity, binding activity, structural molecule activity, enzyme regulator activity, transporter activity, receptor activity, antioxidant activity, nucleic acid binding transcription factor activity, and translation regulator activity [28]. Finally, Galardi et al. performed proteomic profiling by high-resolution MS of exosomes from a primary RB tumor cell line and a primary RB cell line established from vitreous seeds, which resulted in the identification of 3637 proteins [29]. Gene-enrichment analysis of exclusively and differentially expressed proteins and network analysis detected upregulated proteins related to invasion and metastasis involving extracellular matrix remodeling and interaction, resistance to anoikis, and the metabolism of glucose and amino acids in RB vitreous seeds exosomes [29]. Taken together, these proteomic analyses had in common that a more invasive or metastatic RB situation was associated with signaling pathways related to proliferation or inhibition of cell death as well as modulation of the ECM.

Complementary to the previous results, our study identified 4756 proteins by data-independent MS and revealed differences between the etoposide-sensitive WERI-RB1 and the etoposide-resistant WERI-ETOR cell lines. In accordance with other studies, proteins known to be associated with aggressiveness in other tumors, e.g., JAM2 (junctional adhesion molecule-A) [30], were found differentially expressed. Interestingly, pathway analysis revealed induction of, e.g., the “retinoid metabolism and transport” pathway in WERI-ETOR cells. This result is consistent with a study by Nicoud et al., which demonstrated that subretinal delivery of Y79 RB cell-infected retinoid-binding protein gene promoters led to the formation of photoreceptor cells in the mouse retina [31]. In line with this, Vene et al. showed that N-(4-hydroxyphenyl) retinamide inhibited RB tumor growth in a mouse model in vivo and induced cell death in Y79 RB cells in vitro [32]. Khanna et al. detected that retinoic acid upregulates neural retina leucine zipper, a transcription factor expressed in photoreceptors in Y79 [33]. In contrast, Kyritsis et al. have demonstrated an inhibitory effect of retinol and retinoic acid on the RB cell line Y79 [34].

Interestingly, as constituents of the “retinoid metabolism and transport” network, we also identified the heparan sulfate proteoglycans (HSPGs) and membrane-linked ECM receptors glypican-2 and -6 as well as syndecan-1, -2, and -4 downregulated in the WERI-ETOR cell line. As components of the tumor microenvironment, ECM proteins play a critical role in tumor growth and metastasis [35]. In this regard, we previously observed an extensive remodeling of various ECM components in the WERI-ETOR compared to the WERI-RB1 cell line, indicating that the ECM also plays a role in mediating chemotherapy-resistance formation of RB [15]. HSPGs are key players in various processes during neural development and malignant situations [35,36]. They preferentially bind to the basal lamina of blood vessels as well as to ependymal and endothelial surfaces, where they act as disposal sites for a variety of cytokines and growth factors [37]. Particularly, syndecan-1 and -2 have been described to influence VEGF-dependent neovascularization, which is an important mechanism to influence RB growth [38,39,40]. Lau et al. observed a reduced glypican-6 mRNA level in RB associated with non-random allelic loss at 13q31 that could contribute to the development of RB [41].

Furthermore, we observed an activation of the “sphingolipid de novo biosynthesis” pathway in WERI-RB1, which could most likely induce apoptosis and therefore, lead to an etoposide vulnerability. Several studies already noted the effect of sphingolipids on cancer [42]. A previous study by our group revealed a functional association between the sphingolipid metabolism and WERI-ETOR resistance, consistent with the results from our current proteomics study [14]. After exposure to etoposide, sphingosine, an apoptotic mediator, was upregulated in WERI-RB1 and WERI-ETOR, but only in WERI-ETOR, the anti-apoptotic mediator sphingosine-1-phosphate was upregulated [14]. Additionally, Kim et al. noted that exposure to bisphenol A alters transcriptomic and proteomic dynamics in the RB cell line Y79 [43].

According to these data of pathway activation, we can infer that both “retinoid metabolism and transport” and “sphingolipid de novo biosynthesis” may play an important role during therapy resistance in RB. Our results are in line with these studies, which also observed the promotion of retinal cell genesis and may increase the resistance of the WERI-ETOR.

The most relevant finding in the clinical context was the similar MOA of topoisomerase inhibitors but the different MOA of ATPase inhibitors, acetylcholine receptor antagonists, and VEGFR inhibitors compared to WERI-ETOR. Hence, these findings suggest that biochemical processes of topoisomerase inhibitors as well as WERI-ETOR are likely to share the same “positive” molecules, including their expression data. It may, therefore, be the case that etoposide does not affect WERI-ETOR cell populations and, thus, induce no therapeutic activity. Furthermore, and for the same reasons, we speculate that ATPase inhibitors, acetylcholine receptor antagonists, and/or VEGFR inhibitors may be more effective treatment options for etoposide-resistant RB. To develop a complete picture of new treatment strategies in RB, additional studies will be needed that investigate WERI-ETOR and WERI-RB1 using drug classes, as suggested above.

Nevertheless, our study had some limitations. Our in vitro cell model only partially mimics chemotherapy resistance in RB in vivo. Therefore, to further test our hypothesis regarding the potential mechanism of resistance in RB, experiments in an animal model may be promising as a next step. Also, the pathway, as well as the MOA enrichment analysis, were only examined under untreated conditions for WERI-RB1 and WERI-ETOR. Along these lines, future experiments should investigate how both cell lines respond to recommended alternative treatment options.

## 4. Materials and Methods

### 4.1. Cell Culture

The RB cell lines, WERI-RB1 and WERI-ETOR, were cultivated, as previously described [13,14,15,16,17]. The identities of these RB cell lines were verified by DNA fingerprinting and profiling with eight different and highly polymorphic short tandem repeat (STR) loci (Leibniz-Institute DSMZ GmbH, Braunschweig, Germany). The generated STR profiles of the cell lines confirmed a full match of the respective parental reference STR profiles as determined by searching the following cell bank databases: ATCC (Manassas, VA, USA), JCRB (Osaka, Japan), RIKEN (Ibaraki, Japan), KCLB (Seoul, Korea), and DSMZ (Braunschweig, Germany). The purity of both cell lines was demonstrated by analysis of mitochondrial DNA sequences from mouse, rat as well as Chinese and Syrian hamster cells. Analyses with a detection limit of 1:10^5^ showed the absence of mitochondrial sequences from foreign species.

### 4.2. Cell Preparation and Mass Spectrometry

Mass spectrometric analysis was performed, as described elsewhere [44]. Briefly, the Pierce^TM^ Mass Spec Sample Prep Kit for Cultured Cells (Thermo Scientific, Waltham, MA, USA) was used for the preparation and precipitation of peptides according to the manufacturer’s instructions. Cell lysis buffer was used to first lyse 10^6^ cells (n = 5/group), and DNA and RNA were enzymatically digested using nuclease for cell lysis. Total protein concentration was subsequently determined using the fluorescence-based EZQ^TM^ Protein Quantitation Kit (Life Technologies, Carlsbad, CA, USA) according to the manufacturer’s protocol. Briefly, protein samples were spotted, fixed onto an assay paper, and then stained with the proprietary fluorescence reagent. Fluorescence visualization was done with the Typhoon^TM^ FLA 9000 laser scanner (GE Healthcare, Chicago, IL, USA). Subsequently, 50 µg proteins were reduced, alkylated, and precipitated. Enzymatic protein digestion was then carried out by adding the digestion buffer and Lys-C protease to the acetone-precipitated protein pellet. The samples were then frozen at −80 °C with trypsin storage solution to stop digestion.

The samples were lyophilized, resuspended in solvent A (0.1% FA) to a final concentration of 1 µg/mL, and were loaded into the HPLC (Thermo Scientific, Waltham, MA, USA). The reconstituted peptides were first desalted on a trap column (Luna, 5 μm C18 (2), 20 × 0.3 cm; Phenomenex, Torrance, CA, USA) and separated on an analytical column (LC Column, 3 μm C18 (2), 150 mm × 0.3 mm, Phenomenex, Torrance, CA, USA) using a multi-step gradient of solvent B (0.1% FA in ACN) in solvent A for 90 min at a flow rate of 5µL/min. Mass spectra were acquired on a Triple ToF 5600+ (ScieX, Framingham, MA, USA) in a data-independent (SWATH) acquisition mode. The working parameters of the MS were ion spray voltage floating at 5000 V; ion source gas 1, 15; ion source gas 2, 0; curtain gas, 30; source temperature, 0 °C. The optimized declustering potential (DP) was set at 100 V. The SWATH acquisition parameters were as follows: one 0.49965 s MS scan (*m*/*z* 400–1250), followed by 100 variable Q1 windows with the size range 4.6–40.9 Da with 1 Da overlap.

### 4.3. SWATH Data and Quantitative Data Processing

The raw SWATH files were processed using the software tool DIA-NN v1.7.16 (data-independent acquisition by neural networks) developed by Vadim Demichev et al. [24]. The “match between runs” function was used to first develop a spectral library from data-independent acquisition data. The precursor ion-generation settings were set to a peptide length of 7–52 amino acids, a maximum number of missed cleavages to one, and a maximum number of variable modifications to zero. The precursor and fragment ion *m*/*z* range were 350–1250 *m*/*z*. A full analysis log, including the used processing settings, is included in Supplementary Table S3.

Precursors that passed the FDR cutoff of 0.01 were grouped into protein-/gene-groups. The protein groups were reduced to the first listed identifier for those groups consisting of multiple identifiers. The duplicates from a protein group resembling were removed.

The filtered dataset was further processed in the software tool Perseus [45]. MVs were imputed by random numbers drawn from a normal distribution with a width of 0.3 and a downshift of 1.8 (default settings) and total matrix imputation mode. The resulting data matrix was used for subsequent statistical analysis.

MS data have been deposited to the ProteomeXchange Consortium via the PRIDE partner repository [46] with the dataset identifier PXD030924.

### 4.4. Data and Statistical Analysis

Protein data were partly analyzed with the software Omics Playground (BigOmics Analytics, v2.8.5 [47]). Cluster analyses were carried out using the t-distributed stochastic neighbor-embedding (tSNE) algorithm. To increase the statistical reliability of the protein-expression differences, we performed the analysis using commonly accepted methods in the literature, including Welch *t*-test, limma, edgeR as well as DESeq2, and merged the results (meta.q value corresponds to the highest *p*-value derived from the used methods). Significant differential protein expression was considered at a q-value of <0.05 and a logarithmic fold-change (log2FC) of |>2|. Additionally, a variable importance score for each protein was calculated using multiple machine-learning algorithms, including LASSO [48], elastic nets [49], random forests [50], and extreme gradient boosting [51]. An aggregated score was computed and evaluated as the cumulative rank of the variable importance of the different algorithms. The top features were characterized by the highest cumulative ranks. The findings of the machine-learning algorithms were used to validate our marker panels derived from classical statistical tests.

### 4.5. Pathway Enrichment and Drug-Connectivity Correlation

Pathway enrichment was performed using the REACTOME-pathway resource (www.reactome.org, [52]) to understand high-level protein functions and to link information from large molecular data sets. The REACTOME camera workflow, with a focus on metabolic pathways, was used to analyze the function of all quantitative data. Protein signatures were further correlated to known drug profiles from the L1000 database [53] by running the gene set enrichment analysis (GSEA) algorithm [54].

## 5. Conclusions

Our study identified over 4700 proteins in both cell lines examined and further discovered significant differences between WERI-RB1 and WERI-ETOR. In particular, the pathways “retinoid metabolism and transport” and “sphingolipid de novo biosynthesis” seem to play important roles in etoposide resistance, which could further clarify the understanding of resistance in RB. By drug-connectivity analysis using the L1000 database, this study also revealed a similar MOA for topoisomerase inhibitors and WERI-ETOR but a different MOA and potential new treatment options for ATPase inhibitors, acetylcholine receptor antagonists, and VEGFR inhibitors. Taken together, our study, which characterized WERI-RB1 and WERI-ETOR as a cell line model for the human situation in RB, offers new treatment ideas that should be tested in upcoming experiments. Additionally, validation of found differences in protein expression is warranted.

## Figures and Tables

**Table 1:** Top 40 differentially expressed proteins between WERI-RB1 and WERI-ETOR cell lines detected by machine-learning algorithms (LASSO, elastic nets, random forests, extreme gradient boosting). Proteins are sorted according to their fold-change. Log fold-change > 0 represents a higher expression of the protein in the WERI-ETOR group. Significance was calculated using four commonly accepted methods in the literature (Welch *t*-test, limma, edgeR, DESeq2) and merged (meta.q-value).

Gene Symbol	Full Protein Name	Chromosome	Meta.q-Value	Log Fold-Change [WERI-ETOR/WERI-RB1]
DHRS2	Dehydrogenase/reductase SDR family member 2	14	3.051 × 10^−5^	4.062
S100A1	Protein S100-A1	1	1.275 × 10^−6^	3.828
KRT8	Keratin 8	12	6.307 × 10^−4^	3.762
NTM	NAC domain-containing protein 69	11	2.402 × 10^−4^	3.368
EPPK1	Epiplakin	8	1.879 × 10^−4^	2.910
AHNAK	AHNAK nucleoprotein	11	3.570 × 10^−4^	2.708
SEPTIN6	Septin 6	X	1.262 × 10^−4^	2.540
TES	Testin	7	1.615 × 10^−5^	2.513
DIAPH1	Protein diaphanous homolog 1	5	3.634 × 10^−6^	2.174
GNPDA1	Glucosamine-6-phosphate isomerase 1	5	1.576 × 10^−6^	2.158
CRLF3	Cytokine receptor like factor 3	17	5.155 × 10^−4^	1.942
MEX3A	RNA-binding protein MEX3A	1	4.712 × 10^−5^	−1.871
CDH2	Cadherin-2	18	1.581 × 10^−5^	−1.955
TOP2B	DNA topoisomerase 2-beta	3	8.151 × 10^−6^	−2.155
CDK6	Cyclin-dependent kinase 6	7	7.362 × 10^−5^	−2.156
ATP2B1	Plasma membrane calcium-transporting ATPase 1	12	7.630 × 10^−6^	−2.160
TOP2A	DNA topoisomerase 2-alpha	17	6.932 × 10^−7^	−2.178
GNAT2	Guanine nucleotide-binding protein G(t) subunit alpha-2	1	2.064 × 10^−5^	−2.189
SERPINE2	Serpin family E member 2	2	3.716 × 10^−4^	−2.201
OCIAD2	OCIA domain-containing protein 2	4	2.556 × 10^−6^	−2.234
CAMK2D	Calcium-dependent protein kinase II	4	3.350 × 10^−5^	−2.238
CTNNBIP1	Catenin-beta-interacting protein 1	1	4.668 × 10^−3^	−2.408
LMOD1	Leiomodin-1	1	6.385 × 10^−6^	−2.534
ACBD7	Acyl-CoA-binding domain-containing protein 7	10	1.349 × 10^−5^	−2.720
GDAP1L1	Ganglioside-induced differentiation-associated protein 1-like 1	20	5.081 × 10^−5^	−2.734
PLIN2	Perilipin-2	9	1.793 × 10^−5^	−2.769
DPYSL3	Dihydropyrimidinase like 3	5	1.096 × 10^−5^	−2.792
AMPH	D-alanyl-D-alanine-carboxypeptidase/endopeptidase AmpH	7	6.121 × 10^−6^	−2.842
MAP2	Microtubule-associated protein 2	2	3.928 × 10^−5^	−2.966
ATP1B1	Sodium/potassium-transporting ATPase subunit beta-1	1	2.994 × 10^−5^	−3.000
GNGT2	Guanine nucleotide-binding protein subunit gamma-T2	17	8.151 × 10^−6^	−3.000
CRX	Cone-rod homeobox protein	19	1.636 × 10^−4^	−3.177
GAP43	Neuromodulin	3	3.011 × 10^−3^	−3.236
SH3BGRL	SH3 domain-binding glutamic acid-rich-like protein 3	X	5.071 × 10^−5^	−3.325
TAGLN3	Transgelin-3	3	3.634 × 10^−6^	−3.346
ACAA2	3-ketoacyl-CoA thiolase	18	2.119 × 10^−7^	−3.640
BASP1	Brain-acid-soluble protein 1	5	1.879 × 10^−4^	−3.664
RCVRN	Recoverin	17	2.248 × 10^−4^	−3.886
MACROH2A2	Core histone macro-H2A.2	10	1.791 × 10^−5^	−4.777
H1-5	Histone H1.5	6	2.846 × 10^−6^	−7.812

## Data Availability

The mass spectrometry proteomics data have been deposited to the ProteomeXchange Consortium via the PRIDE partner repository [46] with the dataset identifier PXD030924.

## Supplementary Materials

The following supporting information can be downloaded at: https://www.mdpi.com/article/10.3390/ijms23074058/s1.
